# Household access to non-communicable disease medicines during universal health care roll-out in Kenya: A time series analysis

**DOI:** 10.1371/journal.pone.0266715

**Published:** 2022-04-20

**Authors:** Zana Wangari Kiragu, Peter C. Rockers, Monica A. Onyango, John Mungai, John Mboya, Richard Laing, Veronika J. Wirtz

**Affiliations:** 1 Department of Global Health, Boston University School of Public Health, Boston, Massachusetts, United States of America; 2 Innovation for Poverty Action, Nairobi, Kenya; 3 School of Public Health, University of Western Cape, Bellville, South Africa; IGMC: Indira Gandhi Medical College, INDIA

## Abstract

**Objectives:**

This study aims to describe trends and estimate impact of county-level universal health coverage expansion in Kenya on household availability of non-communicable disease medicines, medicine obtainment at public hospitals and proportion of medicines obtained free of charge.

**Methods:**

Data from phone surveillance of households in eight Kenyan counties between December 2016 and September 2019 were used. Three primary outcomes related to access were assessed based on patient report: availability of non-communicable disease medicines at the household; non-communicable disease medicine obtainment at a public hospital versus a different outlet; and non-communicable disease medicine obtainment free of cost versus at a non-zero price. Mixed models adjusting for fixed and random effects were used to estimate associations between outcomes of interest and UHC exposure.

**Results:**

The 197 respondents with universal health coverage were similar on all demographic factors to the 415 respondents with no universal health coverage. Private chemists were the most popular place of purchase throughout the study. Adjusting for demographic factors, county and time fixed effects, there was a significant increase in free medicines (aOR 2.55, 95% CI 1.73, 3.76), significant decrease in medicine obtainment at public hospitals (aOR 0.68, 95% CI 0.47, 0.97), and no impact on the availability of non-communicable disease medicines in households (aβ -0.004, 95% CI -0.058, 0.050) with universal health coverage.

**Conclusions:**

Access to universal health coverage caused a significant increase in free non-communicable disease medicines, indicating financial risk protection. Interestingly, this is not accompanied with increases in public hospitals purchases or household availability of non-communicable disease medicines, with public health centers playing a greater role in supply of free medicines. This raises the question as to the status of supply-side investments at the public hospitals, to facilitate availability of quality-assured medicines.

## Introduction

### Non-communicable diseases in Kenya

Non-communicable diseases (NCDs)—comprising cardiovascular diseases, diabetes, chronic respiratory diseases and cancer—are the leading cause of morbidity and mortality worldwide [1]. The burden of NCDs is on the rise at a disproportionate rate in low and middle income countries (LMICs) [2]. In addition, 80% of NCD-related deaths, occur in the region, with 90% of these deaths being premature [2,3]. This is a concern considering LMIC health systems are already constrained by the demands of infectious diseases [4].

In Kenya, NCDs are responsible for more than 50% of in-patient hospital admissions and 39% of all deaths annually [1,5,6]. Cardiovascular diseases (CVD) account for the majority of NCD-related deaths in the country [2]. Prevalence of NCDs in Kenya varies by region; for instance the prevalence of diabetes is estimated at 10.7% in urban areas and 2.7% in rural areas [1]. Furthermore, one study reported the prevalence of asthma, diabetes and hypertension ranged from 3.0% in Narok County to 30.2% in Kwale County [7]. It is also likely that prevalence of NCDs is underestimated, considering the low level of screening. In the first nationally representative survey for NCD risk factors (2015 STEPwise Survey), 56% of respondents and 88% of respondents reported never having been screened for raised blood pressure and blood sugar respectively [1].

### Availability of NCD medicines in Kenya

Availability of quality assured medicines is a major bottleneck to NCD prevention and control in Kenya [3]. A national survey on the availability of NCD medicines reported that 33% of chronic disease patients in Kenya have their medicines at home [8]. In addition, the 2015 STEPwise survey reported that of those diagnosed with hypertension, only one fifth were taking a medicine prescribed by a health worker, and it is unclear whether these patients self-prescribe or are simply not taking any medication [1]. Factors driving low availability of NCD medicines at households include the challenge of affordability of the medicines. An analysis of affordability of NCD medicines in public and private sector reported that antihypertensive and antidiabetic medications would set back one’s annual income by 1%-2% when purchasing in the public sector and 8%-10% when purchasing in the private sector [2]. About 50% of healthcare is provided by the private sector–where services are more expensive, though more accessible [2].

Another issue is the challenge of medicine stock-outs in the public sector. It is reported that the Kenya Medical Supplies Authority (KEMSA), the primary supplier for the public sector, typically fulfills 70% of the orders requested by counties [9]. Furthermore, availability of NCD medicines in Kenya falls below the WHO recommended 80%, with a study on health facilities in eight counties reporting about 51% availability of NCD medicines in public facilities, 50% in non-profit facilities and 62% in for-profit private chemists [3,10]. In addition, patients report a negative perception of availability of medicines for NCDs in public facilities and thus rely on the private sector where treatment is more expensive [8,11].

NCDs place a continuous social and economic strain on households, in view of their long-term management [1]. Poorer and vulnerable populations disproportionately suffer the consequences of expensive NCD management, as it is the poorest who have the least health insurance coverage and report the highest proportion of catastrophic health expenditures [2,12]. To promote equitable access to preventive and curative health services for NCDs, Universal Health Coverage (UHC) was noted as one of the key elements needed, as detailed in the World Health Organization’s (WHO) Global Action Plan for NCDs [13]. Kenya’s national strategy for prevention and control of NCDs also recognizes this, highlighting an equity-based approach as one of the guiding principles for NCD management [3,6].

### Universal health coverage (UHC) in Kenya

Several efforts have been made in Kenya to shift towards UHC and increase access to health services through expansion of the benefits package of the National Hospital Insurance Fund (NHIF)—the main public health insurance provider in the country [14–16]. There has also been heightened advocacy interventions to increase population subscription to NHIF [14–16]. These changes have increased the demand for health services [15]. Moreover, devolution of the health function to counties has further increased demand and the public’s expectation of improved service provision[15].

UHC is a priority agenda policy of the Kenyan government, in line with SDG 3.8 *to “achieve UHC including financial risk protection*, *access to quality essential healthcare services and access to safe and effective quality and affordable essential medicines and vaccines for all”* [17,18]. The Kenyan government has made the commitment to achieve UHC in the country by 2022 [14,17,19]. The pilot stage of the national UHC scheme *“Afya Care”* was launched in four counties–Isiolo, Kisumu, Machakos and Nyeri—in December 2018, with plans to scale up to the remaining 43 counties by 2022 [14,17,19]. At the County level, *MakueniCare*, a UHC scheme for Makueni County has also emerged [20,21]. A detailed description of these UHC schemes and demographic characteristics of all the counties included in this study can be found in S1 Appendix.

### Study objective

The aim of this study was to describe trends and estimate the impact of County-level expansion of UHC in Kenya using a panel dataset collected from households in eight counties. The primary outcomes of the study are NCD medicines availability, NCD medicine obtainment at a public hospital versus a different outlet, and free obtainment of NCD medicines.

## Methods

### Study design & setting

**Fig 1:**
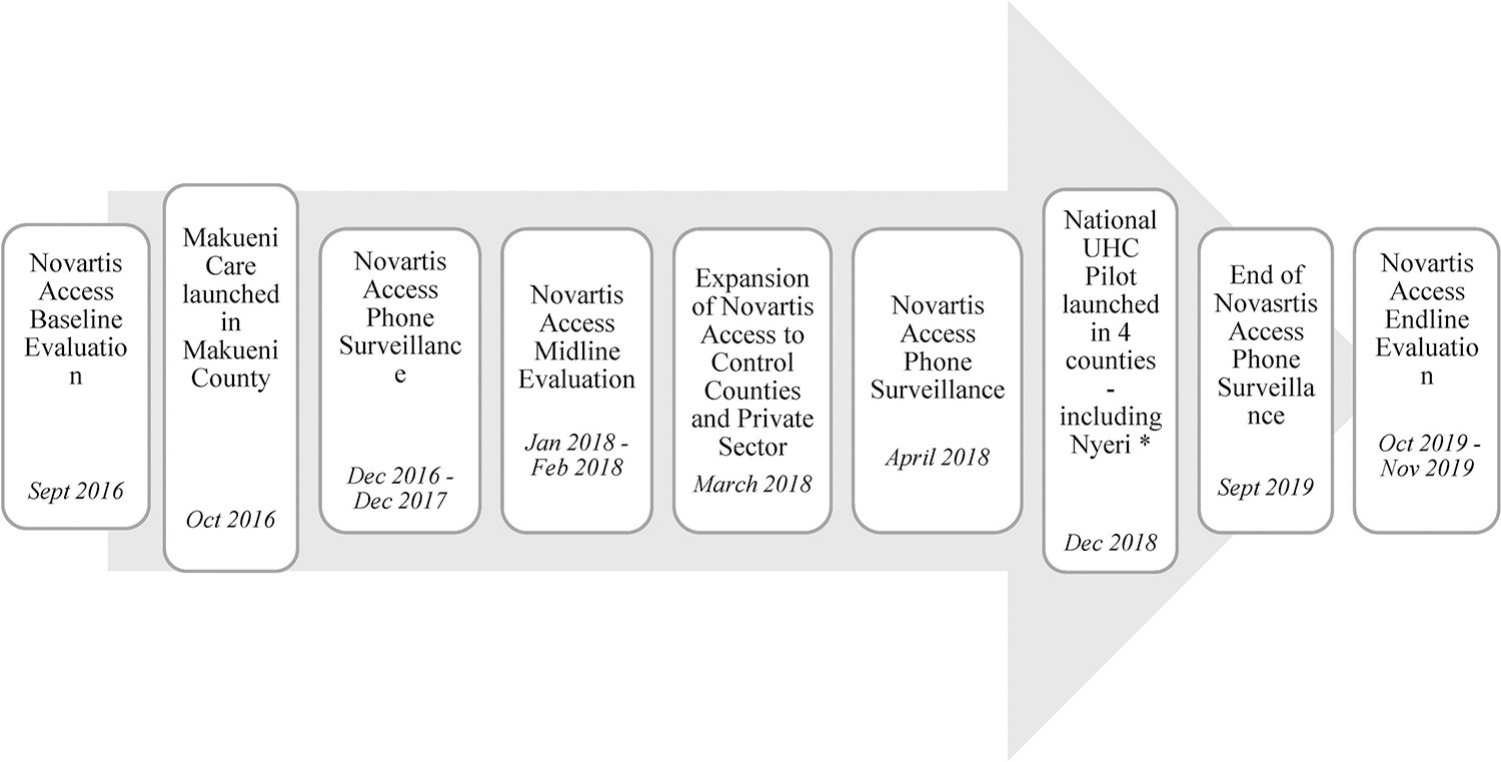
Timeline of events during surveillance. *According to the UHC Coordinator in Nyeri, mass registration for Afya Care was undertaken in the community between November 2018 and February 2019. Registration for UHC in Nyeri was closed in May 2019 when they had reached 88% coverage (Written communication, May 2020).

In 2015, Novartis launched the Novartis Access program in Kenya. The program was designed to offer a basket of 14 medicines for NCDs at a discounted price of $1 per month [22,23]. To evaluate the impact of this program on availability and affordability of medicines, a cluster randomized control study (RCT) was designed, with four counties in the control arm and four counties in the intervention arm [22–24]. The sample size for this cluster RCT was determined through a power analysis described elsewhere [24]. In addition to the in-person survey based evaluation, telephone monitoring took place throughout the study period to measure availability and price of medicines in the household. Telephone surveillance took place for a total of 31 months, starting in December 2016 after Novartis Access Program baseline data collection and continued until December 2017, right before the start of midline data collection. After the randomized control trial ended in February 2018, telephone surveillance resumed, from April 2018 to September 2019, after which the endline evaluation of the program took place (Fig 1). This telephone surveillance aligned with the emergence of UHC schemes in two of the Novartis Access counties. We therefore leveraged an existing longitudinal dataset to explore the impact of these UHC schemes of interest on medicines availability and price.

A rotating one-third sample of these households were surveyed by phone each month, such that a given household received a surveillance call every quarter. Using an abbreviated version of the instruments from baseline evaluation, data was collected via telephone, on the availability of medicines for NCDs–namely asthma, breast cancer, diabetes and cardiovascular disease, the price at which these medicines were purchased, and where they were purchased (public hospital, public health center/clinic, mission hospital/clinic, private for-profit hospital and private chemist).

### Study population

The sampling strategy for the main Novartis Access study from which data for this paper is obtained is described in detail elsewhere [24]. In summary, 10 enumeration areas (EAs) were randomly sampled using probability proportional to size in each of the eight study counties. A random starting point in each EA was selected to begin screening households for eligibility, defined as having an adult aged 18 years or older diagnosed and prescribed medicines for either hypertension, diabetes, asthma, breast cancer, dyslipidemia or heart failure [22,24]. At the start of telephone surveillance, a random sample of 400 of the 639 respondents from baseline data collection were selected for surveillance. After the midline RCT evaluation, all respondents remaining in the study were included in the telephone surveillance calls.

### Universal health coverage

Universal health coverage (UHC) was defined taking into consideration the County and time of implementation of UHC. Two of the eight counties in our study were involved in the special UHC efforts in the country. *MakueniCare* was implemented in Makueni County in October 2016 before the surveillance period, defined as “Always UHC” in this study. The national UHC pilot *Afya Care* was launched in Nyeri in December 2018, defined as “Switched to UHC” in this analysis. The six other counties in the study without these UHC schemes were defined as “Never UHC”.

### Outcomes

The outcomes of interest were NCD medicines availability; NCD medicine obtainment at a public hospital versus a different outlet; and free obtainment of NCD medicines. Availability was defined as having at least one medicine for each of the NCDs reported at baseline available in the household. Among the medicines reported as available (N = 4,646), the place of purchase of the medicine and cost of the total supply was recorded. To define place of purchase, a binary variable was generated for each of the five sectors namely public hospitals (level 4 facilities and above), public health centers and clinics (level 3 and 2 facilities), mission hospitals/clinics, private hospitals and private chemists. Since *MakueniCare* and *Afya Care* were targeted to public hospitals (level 4 and above), the focus was on the binary variable denoting public hospital versus a different outlet. The proportion of medicines obtained for free was a binary variable defined as NCD medicine obtainment free of cost (Kshs 0) versus at a non-zero price. There was 8.3% missing price data.

### Statistical analyses

#### Main analysis

The full surveillance sample was used for the main analysis. Baseline demographic characteristics and outcomes of interest were compared between those with and without UHC exposure. Principal component analysis (PCA) was used for generation of household wealth index at baseline, and has previously been described [25]. For continuous variables, regression adjusting for counties as clusters was used to estimate differences between UHC groups, while Pearson’s Chi-squared tests adjusting for County clusters were undertaken for categorical variables.

STATA’s mixed command was used to estimate unadjusted and adjusted effects for the continuous outcome variable NCD medicines availability. For the categorical outcomes NCD medicine obtainment at a public hospital versus a different outlet and free obtainment of NCD medicines, STATA’s melogit package was used to estimate odds ratios.

#### Sensitivity analysis

The analyses were repeated using only respondents who were in the original surveillance sub-sample selected at baseline. The analyses were also repeated adjusting for assignment to the Novartis Access intervention in the original trial.

#### Validation

A random 10% subsample of the telephone calls in our sample were followed up by an unannounced in-person visit to validate responses between December 2016 and December 2017. A kappa statistic was calculated for medicines availability to determine agreement between the two methods of data collection.

STATA 16.0 was used for all analyses. Charts were generated using Microsoft Excel.

### Ethics

The protocol was approved by the Institutional Review Boards at Maseno University in Kenya and at Boston University Medical Center, USA. A research permit was also obtained from the Kenyan government through the National Commission for Science, Technology and Innovation (NACOSTI). Written informed consent was obtained for all participants at the start of the study.

### Role of the funding source

The funding agreement is publicly available. The funder had no role in study design, data collection and analysis, decision to publish, or preparation of the manuscript. The corresponding author had full access to all of the data in the study and had final responsibility for the decision to submit for publication.

## Results

### Summary of respondent demographics

Of the 400 respondents randomly selected into the surveillance sample, three were not traceable so no data was collected. Another six were not assigned patient identifiers and one respondent had no baseline demographics—they were also excluded. Five additional respondents were reported dead during the first phone call and were excluded, resulting in 385 respondents. Following the end of the RCT in March 2018, all respondents remaining in the study were added to the surveillance sample, and three of these were reported dead during the first phone call, resulting in 612 respondents for analysis (Figure in S1 Fig). Respondents exposed to UHC were similar to those without UHC on all demographic factors (Table 1). Respondents in the surveillance sub-sample were similar to respondents not initially selected for surveillance on all demographic factors except marriage–such that those not included in the original surveillance sub-sample were less likely to be married (Table in S1 Table).

**Table 1:** Summary of baseline demographics by UHC exposure.

	Never UHC N = 415	Switched to UHC N = 101	Always UHC N = 96	p-value*
**Age**	55.6 (16.7)	66.2 (15.9)	62.2 (13.9)	0.068
**Sex**				0.948
**Male**	131(31.6%)	30 (29.7%)	27 (28.1%)	
**Female**	284 (68.4%)	71 (70.3%)	69 (71.9%)	
**Married**	290 (69.9%)	70 (69.3%)	72 (75.0%)	0.583
**Wealth Quintiles**				0.906
**1 (Poorest)**	97 (23.4%)	12 (11.9%)	8 (8.3%)	
**2**	86 (20.7%)	21 (20.8%)	18 (18.8%)	
**3**	73 (17.6%)	18 (17.8%)	34 (35.4%)	
**4**	79 (19.0%)	26 (25.7%)	18 (18.8%)	
**5 (Wealthiest)**	80 (19.3%)	24 (23.8%)	18 (18.8%)	
** Education**				0.645
** Preschool (less than 1 year completed)/None**	127 (30.6%)	26 (25.7%)	9 (9.4%)	
** Primary School (not completed)**	90 (21.7%)	22 (21.8%)	42 (43.8%)	
** Primary school**	82 (19.8%)	25 (24.8%)	30 (31.3%)	
** Secondary school**	77 (18.6%)	23 (22.8%)	13 (13.5%)	
** Higher than secondary school**	35 (8.4%)	5 (5.0%)	2 (2.1%)	
** Vocational School (Post primary)**	4 (1.0%)	0 (0.0%)	0 (0.0%)	
**Hypertension**	268 (64.6%)	81 (80.2%)	79 (82.3%)	0.36
**Heart Failure**	10 (2.4%)	3 (3.0%)	8 (8.3%)	0.11
**Dyslipidemia**	2 (0.5%)	0 (0.0%)	1 (1.0%)	0.74
**Diabetes**	88 (21.2%)	25 (24.8%)	21 (21.9%)	0.838
**Asthma**	102 (24.6%)	12 (11.9%)	11 (11.5%)	0.379

Data are presented as mean (SD) for continuous measures, and n (%) for categorical measures.

*Adjusted for County clusters.

### Trends in availability, location of purchase and number of free medicines over time

**Fig 2:**
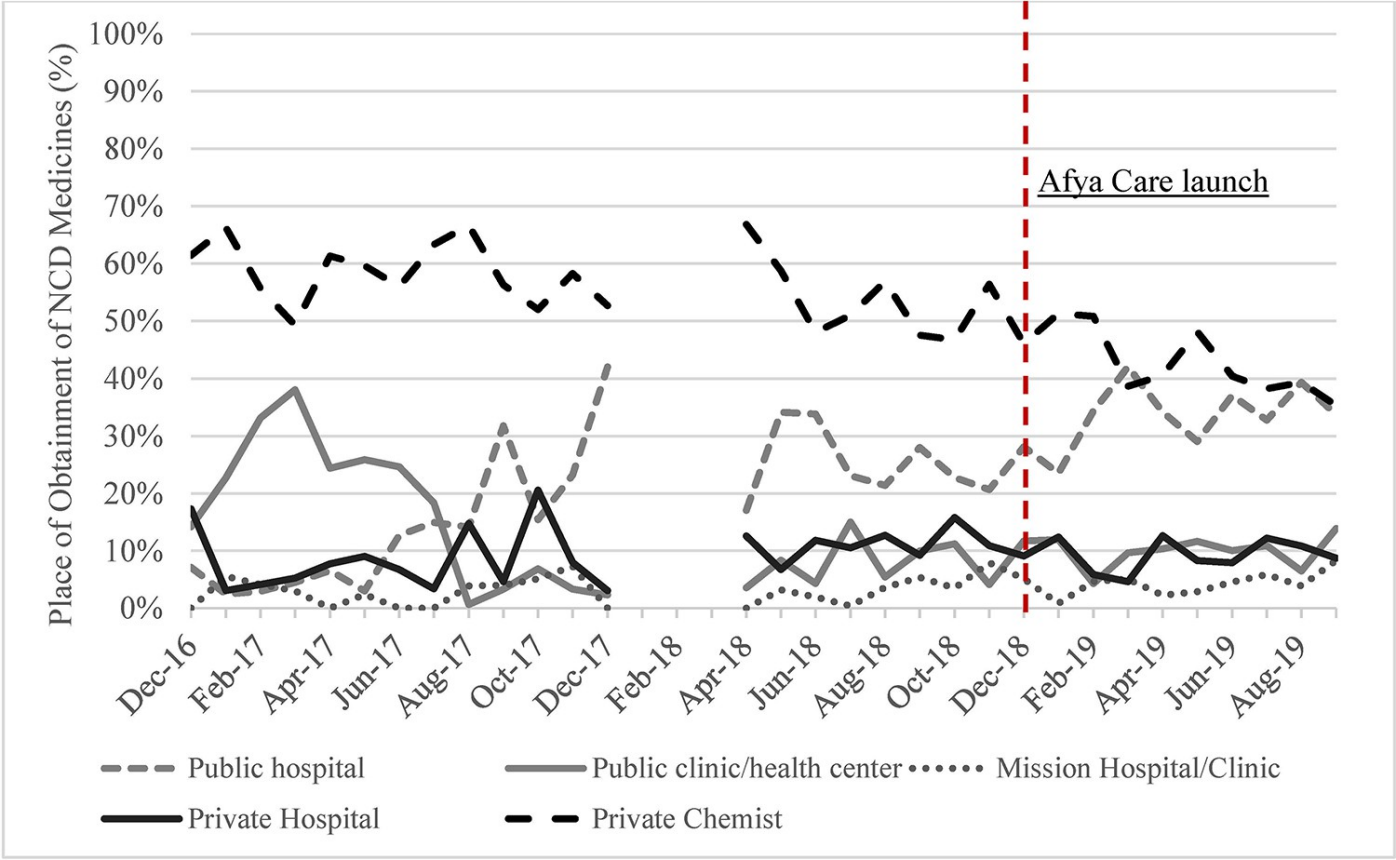
Place of obtainment of NCD medicines.

A very small percentage of purchases, 0.2% (10/6,446), were excluded from the analysis, as they did not occur in any of the five sectors of interest. Throughout the surveillance period, the majority of purchases of NCD medicines were made in private chemists, until the last two months of surveillance where public hospital purchases matched private chemist purchases (Fig 2). On average, 51.2% of purchases of NCD medicines were made at private chemists, 24.2% at public hospitals, 11.4% at public health centers/clinics, 9.6% at private hospitals and 3.6% at mission hospitals/clinics. An increase in NCD purchases in public hospitals, and a decrease in NCD purchases in public health centers/clinics was also observed over time.

**Fig 3:**
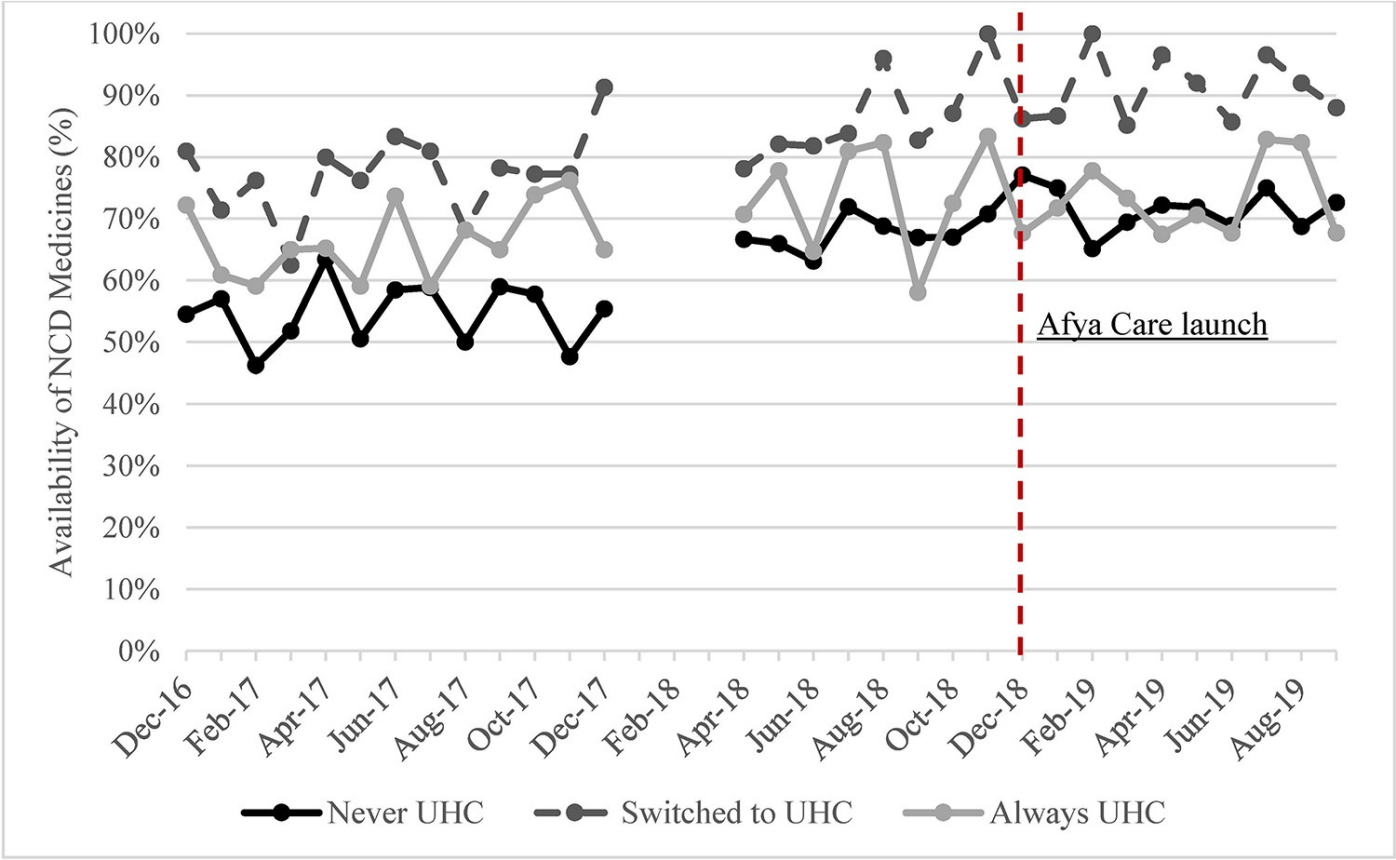
Availability of NCD medicines by UHC.

**Fig 4:**
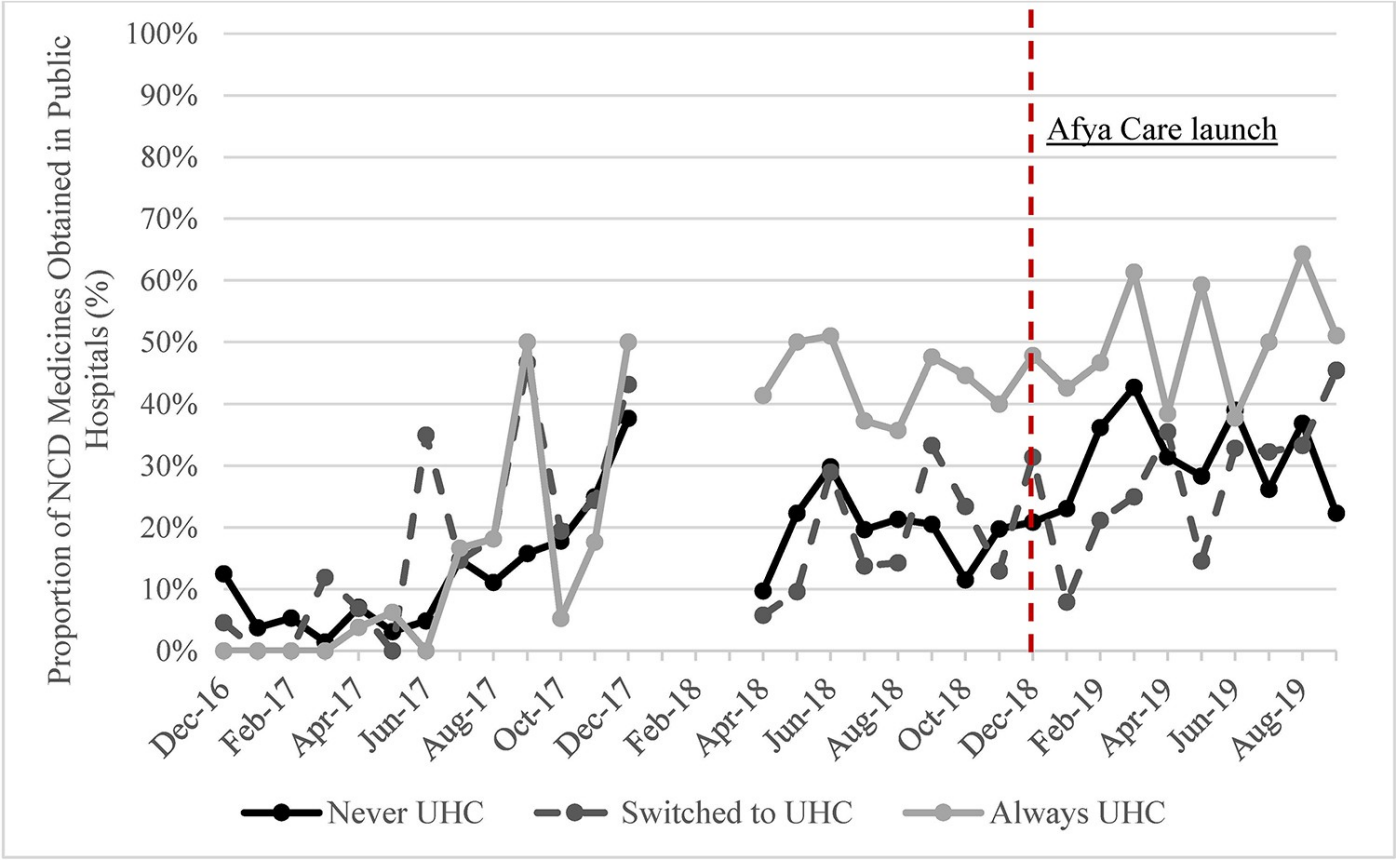
Proportion of NCD medicines obtained in public hospitals by UHC.

**Fig 5:**
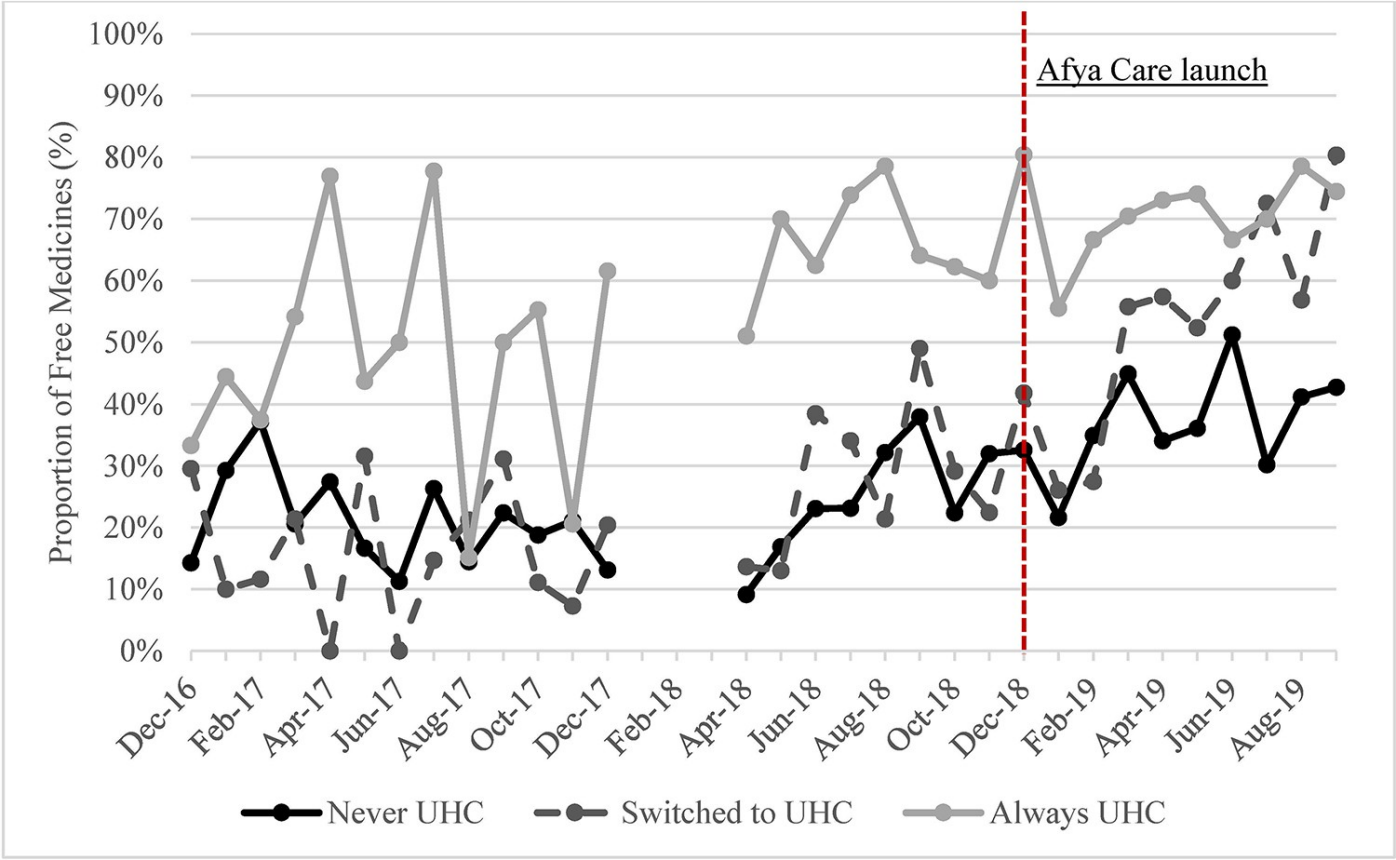
Proportion of NCD medicines obtained free of charge.

The availability of NCD medicines, proportion of NCD medicines obtained in public hospitals and proportion of free medicines increased in all UHC groups over time (Figs 3–5). The steepest increase in proportion of purchases in the public hospitals was observed in the “Always UHC” group relative to the other groups. The steepest increase in the proportion of free medicines purchased was seen in the “Switched to UHC” group. Descriptive graphs for each outcome before and after the launch of Afya Care showed that post-*Afya Care*, the “Switched to UHC” group had a steeper increase in proportion of medicines obtained in public hospitals and proportion of free medicines post Afya-Care launch (see Figure A, B, C, D, E and F in S2 Fig).

### Unadjusted and adjusted effects by exposure to UHC

Unadjusted effects showed no association between UHC and NCD medicines availability or NCD medicines obtainment in public hospitals, though respondents exposed to UHC obtained significantly more medicines free of charge (Table 2). After adjusting for time, County, baseline demographics and baseline NCD diagnosis, there was a significant increased odds of obtaining free NCD medicines with UHC (aOR 2.55, 95% CI 1.73, 3.76). In addition, there was reduced odds of NCD medicine obtainment in public hospitals with exposure to UHC that was borderline significant (aOR 0.68, 95% CI 0.47, 0.97). We conducted a post-hoc power analysis, which accounted for correlation within county and within participants over time and determined that this study is powered to detect an 11-percentage point increase in free NCD medicines, given the observed 27% probability of free medicines in the non-UHC group. We ran an adjusted linear probability model and observed an increase of 15 percentage points in free medicines in the UHC group, corresponding to the adjusted OR of free medicines (2.55). Therefore, we are sufficiently powered at the 5% significance level.

**Table 2:** Effect of UHC on outcomes of interest.

	Unadjusted Effects ^a^		Adjusted Effects ^b^	
	**β (95% CI)**	**p-value**	**β (95% CI)**	**p-value**
**Medicines Available** **(N = 4,747)**	-0.002 (-0.054, 0.051)	0.947	-0.004 (-0.058, 0.050)	0.887
	**Odds Ratio (95% CI)**	**p-value**	**Odds Ratio (95% CI)**	**p-value**
**Medicines Obtained in Public Hospitals** **(N = 6,436)**	0.74 (0.52, 1.07)	0.11	0.68 (0.47, 0.97)	0.04
**Free Medicines** **(N = 5,904)**	3.00 (2.01,4.47)	0.00	2.55 (1.73, 3.76)	0.00

^a^ Unadjusted effects: Adjusted for time as fixed effects and respondent and County as random effects.

^b^ Adjusted effects: Adjusted for time, County, baseline demographics and baseline NCD diagnosis as fixed effects, and respondent and County as random effects.

Results from an analysis including only observations from those in the original surveillance sub-sample (N = 385) gave largely similar results to the main analysis, though it was underpowered to detect changes in the free medicines outcome, thus conclusions on significance were different (see S2 Appendix). Adjusting for assignment to treatment yielded similar results in the unadjusted models (See Table in S2 Table). Validation tests on 2.8% of the full surveillance sample revealed agreement (see Table in S3 Table).

## Discussion

This study provides relevant evidence for policy decisions related to the roll out of UHC. First, our study shows that *Afya Care* and *MakueniCare* have succeeded with decreasing the probability of patients spending on their NCD medicines. Second, this has not yet translated to higher availability of medicines at household level with the UHC-exposed,–suggesting that having medicines in the household is driven by multiple factors beyond affordability. A previous study reported that factors influencing having medicines at home in Kenya included socio-economic status, availability of medicines in the respondent’s community and patient perceptions on medicines [8]. This same study reported no significant difference in availability of NCD medicines with partial payment for medicines but that there were significantly increased odds of having NCD medicines when it was possible to obtain all medicines free of charge [8]. Both *Afya Care* and *MakueniCare* targeted public facilities, where challenges of medicines stock-outs persist. Patients in this study previously reported being able to receive only some of their medicines at no cost in public facilities due to stock-outs, and having to resort to private chemists for the medicines they could not find in the public facilities [11].

Third, our study shows that private chemists were the most popular place of purchase of NCD medicines over time, which is not surprising considering previously published evidence showing availability of NCD medicines in LMICs is higher in the private sector [10,26]. Fourth, while a temporal trend of increasing proportion of NCD medicines obtained in public hospitals was observed, adjustment for time and other factors revealed that UHC exposure significantly reduced the odds of obtaining NCD medicines in public hospitals. This suggests that the observed trends are being driven by another factor (not UHC) which has changed over time, which we have not measured in our study. One explanation for the decreased odds in public hospitals but increased odds of free medicines is that in the “Switched to UHC” group, public health centers/clinics, not hospitals, had a more prominent role in providing free medicines (see Table A, Fig A and B in S3 Appendix). Another possible explanation is tied to the quantity of medicines obtained with the provision of free medicines in public hospitals; in that respondents are able to obtain larger quantities of medicines since they were free of charge, resulting in fewer people obtaining medicines in the public hospitals (see Table in S4 Table).

The steepest trend increase in proportion of NCD medicines obtained in public hospitals was observed in the “Always UHC” group–in Makueni County, where other changes in public healthcare delivery could be driving these observed trends. These include, increase in the number of public facilities which have more than doubled between 2013 and 2018, and increases in quantity and quality of human resource for healthcare delivery [21,27,28].

Our study highlights the importance of aligning interventions to subsidize the cost of medicines in the public sector with supply-side investments, particularly in view of the devolution of healthcare delivery to counties. Some of the challenges that face supply-side investments in the counties include delays with disbursement of funds from the National Treasury and challenges with collecting revenues at the County level [27]. This need for increased supply-side investment has been acknowledged, and Makueni County recently received a Kshs 100 million from the Kenya National Treasury, which will be assigned to KEMSA to facilitate stable procurement of medicines [29].

Finally, the validation of telephone surveillance data with in-person visits revealed it is a reliable method to collect data. It has been predicted that there will be an increase in the number of mobile phone subscriptions in Sub-Saharan Africa to match the population of the region by 2020 [30]. The use of mobile phones to collect data is therefore a feasible novel method of surveillance. Mobile phone surveys are useful for their time efficient and cost efficient nature [30,31]. Previous research has reported the utility of mobile surveys for NCD surveillance, particularly to track incidence and prevalence of NCDs in Sub-Saharan Africa in view of the rising burden of disease [31]. Our study is unique in that, as far as we are aware, it is the first of its kind to demonstrate utility of mobile phone surveys in monitoring household availability, location of purchase and number of free purchases of NCD medicines over time. This method of data collection provides a convenient, efficient and reliable way to generate quality evidence to monitor and evaluate existing policy and practices or to formulate new policies [31].

Because of its longitudinal nature, this study provides strong evidence for reduced cost burden on NCD patients with UHC roll-out. However, there are some limitations. The study did not take into account other forms of insurance beyond the special UHC schemes when defining UHC exposure. For instance, Embu County, classified as having no UHC (comparison group), accounts for 32% of individuals with all types of health insurance in the country; the second highest after Nairobi [14]. Therefore, the comparison group may not be a true counterfactual, with selection bias and unknown confounders persisting as a threat to internal validity. Secondly, the definition of health insurance exposure in this analysis is based on County of residence, and respondents were not asked whether they had registered for *AfyaCare* or *MakueniCare* as part of the survey, as this was not the initial goal of surveillance. It is likely that coverage of these schemes is not 100% thus not everyone in our sample may have access to the benefits associated with these schemes. There is a paucity of reliable data on the number of registrants to these schemes. The Nyeri UHC coordinator reported 88% coverage as of May 2019 (Written Communication, May 2020), and *MakueniCare* coverage as of June 2019 can be estimated at approximately 45% [20,32]. Furthermore, results from the regression analysis are largely being driven by observations in Nyeri County, by virtue of the fact that the County-health insurance scheme in Nyeri was initiated during our surveillance period, giving us pre and post UHC data for this County. It is therefore difficult to generalize the findings from the analysis, as there may be other factors unique to Nyeri driving these findings. In line with this, we cannot rule out residual confounding by unmeasured factors that may be driving the outcomes observed.

## Conclusion

This study provides evidence that there is an increase in obtaining free NCD medicines in counties with UHC schemes targeting public hospitals, where NCD diagnosis primarily occurs in Kenya. However, a corresponding increase in obtaining NCD medicines in public hospitals and increase in medicine availability at the household level with UHC was not detected overall. In fact, the opposite was observed, and there was a decrease in proportion of NCD medicines obtained in public hospitals purchases with UHC exposure. Our findings suggest that the increasing proportion of free NCD medicines observed were largely obtained in public health centers/clinics. While these results are limited with regard to generalizability, the lessons learned are important and suggest that increased investment into the supply-side of health delivery, to minimize medicine stockouts could support the efforts to subsidize health care delivery and likely increase their impact. The data from this study was collected using telephone surveillance, which is increasingly emerging as a convenient, efficient and reliable means of data collection in Sub-Saharan Africa. Further research is needed to evaluate the impact and sustainability of *Afya Care* and *MakueniCare* and alignment of these initiatives with NHIF, to facilitate Kenya’s move towards UHC.

### Data sharing

Data underlying the study cannot be made publicly available due to ethical restrictions, as public availability of the data in its present form could compromise participant’s privacy and anonymity. However, researchers may request data for baseline and surveillance from Boston University (https://sites.bu.edu/evaluatingaccess-novartisaccess/kenya/data/).

## Supporting information

S1 Fig Surveillance sample description. (DOCX)

S2 Fig Descriptive graphs pre and post *Afya-Care* launch. (DOCX)

S1 Table Summary of characteristics of surveillance sample. (DOCX)

S2 Table Effect of UHC on outcomes of interest adjusting for assignment to Novartis Access Intervention in the original trial. (DOCX)

S3 Table Validity of phone interviews. (DOCX)

S4 Table Quantity of medicine obtained for select medicines. (DOCX)

S1 Appendix Description of counties and UHC schemes in Kenya. (DOCX)

S2 Appendix Effect of UHC on outcomes of interest for the surveillance sub-sample selected at baseline. (DOCX)

S3 Appendix Proportion of NCD medicines obtained in public health centers/clinics. (DOCX)
