# Supplementary material for: Household access to non-communicable disease medicines during universal health care roll-out in Kenya: A time series analysis
Source: PLoS One. 2022 Apr 20;17(4):e0266715. doi: 10.1371/journal.pone.0266715 (PMC9020677; doi:10.1371/journal.pone.0266715)
Supplement: S1 Fig — (DOCX) [file pone.0266715.s001.docx]

**S1 Fig: Surveillance sample description**
