# Supplementary material for: Household access to non-communicable disease medicines during universal health care roll-out in Kenya: A time series analysis
Source: PLoS One. 2022 Apr 20;17(4):e0266715. doi: 10.1371/journal.pone.0266715 (PMC9020677; doi:10.1371/journal.pone.0266715)
Supplement: S2 Fig — (DOCX) [file pone.0266715.s002.docx]

**S2 Fig: Descriptive graphs pre and post *Afya-Care* launch**

**Fig A: Availability of medicines by UHC pre-*Afya Care* launch**

**Fig B: Availability of medicines by UHC post-*Afya Care* launch**

**Fig C: Proportion of NCD medicines obtained in public hospitals pre-*Afya Care* launch**

**Fig D: Proportion of NCD medicines obtained in public hospitals post-*AfyaCare* launch**

**Fig E: Proportion of free medicines by UHC pre-*Afya Care* launch**

**Fig F: Proportion of free medicines by UHC post-*Afya Care* launch**
