# Supplementary material for: Household access to non-communicable disease medicines during universal health care roll-out in Kenya: A time series analysis
Source: PLoS One. 2022 Apr 20;17(4):e0266715. doi: 10.1371/journal.pone.0266715 (PMC9020677; doi:10.1371/journal.pone.0266715)
Supplement: S1 Table — (DOCX) [file pone.0266715.s003.docx]

**S1 Table: Summary of characteristics of surveillance sample**

|  | Total Baseline Sample | Excluded from Surveillance at Baseline | Surveillance Sub Sample at Baseline | p-value* |
| --- | --- | --- | --- | --- |
|  | **N=639** | **N=249** | **N=390** |  |
| Age | 58.5 (16.8) | 59.2 (16.7) | 58.1 (16.8) | 0.316 |
|  |  |  |  |  |
| Sex |  |  |  | 0.789 |
| Male | 199 (31.1%) | 74 (29.7%) | 125 (32.1%) |  |
| Female | 440 (68.9%) | 175 (70.3%) | 265 (67.9%) |  |
|  |  |  |  |  |
| Married | 446 (69.8%) | 154 (61.8%) | 292 (74.9%) | 0.0005 |
|  |  |  |  |  |
| Wealth Quintiles |  |  |  | 0.815 |
| 1 (Poorest) | 126 (19.7%) | 59 (23.7%) | 67 (17.2%) |  |
| 2 | 129 (20.2%) | 44 (17.7%) | 85 (21.8%) |  |
| 3 | 129 (20.2%) | 45 (18.1%) | 84 (21.5%) |  |
| 4 | 128 (20.0%) | 60 (24.1%) | 68 (17.4%) |  |
| 5(Wealthiest) | 127 (19.9%) | 41 (16.5%) | 86 (22.1%) |  |
|  |  |  |  |  |
| Education |  |  |  | 0.953 |
| Preschool (less than 1 year completed)/None | 172 (26.9%) | 74 (29.7%) | 98 (25.1%) |  |
| Primary School (not completed) | 162 (25.4%) | 61 (24.5%) | 101 (25.9%) |  |
| Primary school | 140 (21.9%) | 54 (21.7%) | 86 (22.1%) |  |
| Secondary school | 116 (18.2%) | 38 (15.3%) | 78 (20.0%) |  |
| Higher than secondary school | 45 (7.0%) | 22 (8.8%) | 23 (5.9%) |  |
| Vocational School (Post primary) | 4 (0.6%) | 0 (0.0%) | 4 (1.0%) |  |
| Hypertension | 445 (69.6%) | 177 (71.1%) | 268 (68.7%) | 0.828 |
| Heart Failure | 24 (3.8%) | 7 (2.8%) | 17 (4.4%) | 0.433 |
| Dyslipidemia | 4 (0.6%) | 2 (0.8%) | 2 (0.5%) | 0.650 |
| Diabetes | 142 (22.2%) | 52 (20.9%) | 90 (23.1%) | 0.634 |
| Asthma | 129 (20.2%) | 52 (20.9%) | 77 (19.7%) | 0.896 |

*Data are presented as mean (SD) for continuous measures, and n (%) for categorical measures.*

**Adjusted for County clusters*
