# Supplementary material for: Household access to non-communicable disease medicines during universal health care roll-out in Kenya: A time series analysis
Source: PLoS One. 2022 Apr 20;17(4):e0266715. doi: 10.1371/journal.pone.0266715 (PMC9020677; doi:10.1371/journal.pone.0266715)
Supplement: S2 Table — (DOCX) [file pone.0266715.s004.docx]

**S2 Table: Effect of UHC on Outcomes of Interest adjusting for assignment to Novartis Access Intervention in the Original Trial**

|  | Unadjusted Effects ^a^ | |
| --- | --- | --- |
|  | **β (95% CI)** | **p-value** |
| Medicines Available  (N=4,747) | -0.0003 (-0.53, 0.053) | 0.99 |
|  | **Odds Ratio (95% CI)** | **p-value** |
| Medicines Obtained in Public Hospitals  (N=6,436) | 0.73 (0.51, 1.05) | 0.09 |
| Free Medicines  (N=5,904) | 3.00 (2.01,4.47) | 0.00 |

**^a^ Unadjusted effects: adjusted for time and Novartis Access assignment as fixed effects and respondent and County as random effects**

**Note: We could not control for assignment to Novartis Access intervention in the adjusted models, due to collinearity with the County variable. This is because assignment to the Novartis Access intervention was made by County in the original trial.**
