# Supplementary material for: Household access to non-communicable disease medicines during universal health care roll-out in Kenya: A time series analysis
Source: PLoS One. 2022 Apr 20;17(4):e0266715. doi: 10.1371/journal.pone.0266715 (PMC9020677; doi:10.1371/journal.pone.0266715)
Supplement: S3 Table — (DOCX) [file pone.0266715.s005.docx]

**S3 Table: Validity of phone interviews**

|  | Telephone Calls | In-Person Visits |
| --- | --- | --- |
| Number of Surveys (N) | 131 | 131 |
| Agreement on availability of medicines reported | Percentage Agreement = 90.8%  Kappa = 0.78 ,95% CI 0.66 – 0.90, p-value =0.00 | |
