# Supplementary material for: Household access to non-communicable disease medicines during universal health care roll-out in Kenya: A time series analysis
Source: PLoS One. 2022 Apr 20;17(4):e0266715. doi: 10.1371/journal.pone.0266715 (PMC9020677; doi:10.1371/journal.pone.0266715)
Supplement: S4 Table — (DOCX) [file pone.0266715.s006.docx]

**S4 Table: Quantity of medicine obtained for select medicines**

|  | Metformin 500mg | | Hydrochlorothiazide 50mg | | Glibenclamide 5mg | |
| --- | --- | --- | --- | --- | --- | --- |
|  | **Paid For (N=299)** | **Free (N=206)** | **Paid For**  **(N=444)** | **Free**  **(N=311)** | **Paid For**  **(N=167)** | **Free**  **(N=145)** |
| Mean number of tablets obtained | 29.8 | 45.0 | 22.8 | 28.1 | 27.8 | 36.2 |
