# Supplementary material for: Household access to non-communicable disease medicines during universal health care roll-out in Kenya: A time series analysis
Source: PLoS One. 2022 Apr 20;17(4):e0266715. doi: 10.1371/journal.pone.0266715 (PMC9020677; doi:10.1371/journal.pone.0266715)
Supplement: S1 Appendix — (DOCX) [file pone.0266715.s007.docx]

**S1 Appendix: Description of counties and UHC schemes**

**Table A: Key characteristics of counties**

| **County** | **Urban Population** [1,2] | **Poverty Headcount** [3–10] | **Proportion of Public Health Facilities***[11] | **Proportion of Population 15 years and older who are literate** [12] |
| --- | --- | --- | --- | --- |
| Narok | 8.7% | 22.1% | 56.1% | 69.3% |
| Kwale | 14.6% | 41.1% | 66.8% | 67.7% |
| Embu | 12.5% | 28.3% | 46.8% | 86.1% |
| Nyeri | 19.9% | 15.5% | 32.8% | 92.7% |
| Kakamega | 9.9% | 33.3% | 54.7% | 80.6% |
| West Pokot | 5.1% | 57.3% | 79.4% | 61.5% |
| Makueni | 7.8% | 30.7% | 67.4% | 82.3% |
| Samburu | 15.2% | 60.1% | 61.5% | 40.3% |

*data extracted on January 21^st^ 2022 from the Kenya Master Health Facility List, where we defined public health facilities as those belonging to the ownership category “Ministry of Health”.

**Afya Care in Nyeri County**

*Afya Care* builds on the national government provision for free care at primary public health facilities (level 2 and 3), and provides for free health services at level 4 and 5 public health facilities. The program was designed to cover a range of curative, preventive and rehabilitative services including free diagnostic services for minor ailments and free medicines [13,14]. To support *Afya Care*, the government set aside USD 40-45 million in funds, to be sourced from taxes, funds reallocated from other ministries and external donors [14]. Funds were allocated to pilot counties to cover operation and maintenance of level 4 and 5 public facilities, public service functions including surveillance and quality control. In addition, KEMSA was also allocated funds for supply of medicines and technologies at secondary care facilities in pilot counties [15].

The *Afya Care* pilot ended in March 2020, and while some lessons learned have been mentioned in news reports, it is unclear if a formal assessment of the pilot will be released [16–18]. The pilot counties have reported some challenges, and the Nyeri County government recently announced discontinuation of the *Afya Care* scheme due to lack of funds [17,19]. Nonetheless, the Ministry of Health is proceeding with scale-up as planned, as evidenced by signing of inter-governmental participation agreements (IPA) with other counties [18,20].

Nyeri County was selected as one of the *Afya Care* pilot counties, alongside Isiolo, Kisumu, Machakos in view of its high burden of non-communicable diseases [21,22]. It is home to 759,164 people, and has a human development index of 0.59, the second highest rating after Nairobi County with regard to education, health and standards of living [23]. It is largely rural, with clusters of settlements around urban and agricultural centers [22,24]. It also has the highest health facility density in Kenya, at 4.3 facilities per 10,000 people, and more advanced medical infrastructure relative to neighboring counties, translating to medical tourism [25,26].

**MakueniCare in Makueni County**

*MakueniCare* was launched by the Makueni County Government in October 2016 to provide universal access to healthcare for Makueni residents [27]. The scheme leveraged off the existing national policy of free healthcare at primary healthcare facilities (level 2 and 3), and provided for free inpatient and outpatient care for essential curative, preventive and rehabilitative services at the level 4 and 5 public facilities in Makueni County for Makueni residents [15,28]. To achieve this, the County projected that Kshs 200 million would be required. They set an annual premium of Kshs 500/family to generate revenue for the fund [28]. Registration with the scheme was manual initially, making it difficult to track annual membership continuation for the first three years of the scheme. An impact evaluation of MakueniCare is currently underway [15,28]. Makueni County has a population of 987,653 people, with approximately 30% of the population living below the poverty line [28,29]. The county is largely rural, with a health facility density of 3.3 per 10,000 population [25,28].

**References**

1. Kenya National Bureau of Statistics. 2019 Kenya Population and Housing Census Volume 1: Population by County and Sub-County. 2019 Kenya Population and Housing Census. 2019. Available: https://www.knbs.or.ke/?wpdmpro=2019-kenya-population-and-housing-census-volume-i-population-by-county-and-sub-county

2. openAFRICA. 2019 Kenya Population and Housing Census - Distribution of Urban Population by Sex and County . 11 Mar 2020 [cited 25 Jan 2022]. Available: https://open.africa/dataset/2019-kenya-population-and-housing-census/resource/a0437750-81a4-4c99-8a4a-d7b95f534a4f?view_id=11aab149-c449-4949-a90f-2e201f602cc8

3. The Open Institute and The World Bank. Kwale County Factsheet - Open County. 2015 [cited 25 Jan 2022]. Available: https://opencounty.org/factsheet.php?com=9&cid=2

4. The Open Institute and The World Bank. Narok County Factsheet - Open County. 2015 [cited 25 Jan 2022]. Available: https://opencounty.org/factsheet.php?com=9&cid=33

5. The Open Institute and The World Bank. Makueni County Factsheet - Open County. 2015 [cited 25 Jan 2022]. Available: https://opencounty.org/factsheet.php?com=9&cid=17

6. The Open Institute and The World Bank. West-Pokot County Factsheet - Open County. 2015 [cited 25 Jan 2022]. Available: https://opencounty.org/factsheet.php?com=9&cid=24

7. The Open Institute and The World Bank. Samburu County Factsheet - Open County. 2015 [cited 25 Jan 2022]. Available: https://opencounty.org/factsheet.php?com=9&cid=25

8. The Open Institute and The World Bank. Kakamega County Factsheet - Open County. 2015 [cited 25 Jan 2022]. Available: https://opencounty.org/factsheet.php?com=9&cid=37

9. The Open Institute and The World Bank. Nyeri County Factsheet - Open County. 2015 [cited 25 Jan 2022]. Available: https://opencounty.org/factsheet.php?com=9&cid=19

10. The Open Institute and The World Bank. Embu County Factsheet - Open County. 2015 [cited 25 Jan 2022]. Available: https://opencounty.org/factsheet.php?com=9&cid=14

11. Ministry of Health - Republic of Kenya. Kenya Master Health Facility List: Find all the health facilities in Kenya. 2022 [cited 21 Jan 2022]. Available: http://kmhfl.health.go.ke/#/facility_filter/results?county=0a629644-41eb-44b8-a004-e56f06b3c006&operation_status=ae75777e-5ce3-4ac9-a17e-63823c34b55e&owner_type=6a833136-5f50-46d9-b1f9-5f961a42249f

12. Kenya National Bureau of Statistics (KNBS). Kenya Integrated Household Budget Survey (KIHBS) - Basic Report 2015/2016. Nairobi, Kenya; 2018.

13. Kahongeh J. Universal Health Coverage: What you need to know - Daily Nation. Dec 2018. Available: https://nation.africa/kenya/news/universal-health-coverage-what-you-need-to-know-118346?view=htmlamp. Accessed 15 Jul 2020.

14. Nzwili F. Kenyan President Launches Benchmark Universal Health Coverage Pilot, To Become Nationwide In 18 Months - Health Policy Watch. 2018 [cited 26 May 2020]. Available: https://healthpolicy-watch.org/kenyan-president-launches-benchmark-universal-health-coverage-pilot-to-become-nationwide-in-18-months/

15. Dutta A, Mainia T, Ginivan M, Koseki S. Kenya Health Financing System Assessment, 2018:Time to Pick the Best Path. Washington, DC; 2018.

16. Ministry of Health. UHC Pilot expands health services to 3.2 Kenyans. 2019 [cited 28 Aug 2020]. Available: https://www.health.go.ke/uhc-pilot-expands-health-services-to-3-2-kenyans/

17. Mugo I. Counties carry heavy burden as UHC pilot phase comes to an end - Daily Nation. Mar 2020. Available: https://nation.africa/kenya/counties/nyeri/UHC-pilot-phase-comes-to-an-end/1954190-5504216-ihdqh0/index.html. Accessed 15 Jul 2020.

18. Ministry of Health. UHC roll out on course as more governors turn up to sign IPAs. 2020 [cited 28 Aug 2020]. Available: https://www.health.go.ke/uhc-roll-out-on-course-as-more-governors-turn-up-to-sign-ipas/

19. Mugo I. Nyeri suspends free health programme | Nation. 15 Aug 2020. Available: https://nation.africa/kenya/counties/nyeri/nyeri-suspends-free-health-programme-1919466?view=htmlamp. Accessed 28 Aug 2020.

20. Ministry of Health. CS health signs UHC scale up agreement with governors – MINISTRY OF HEALTH. 2020 [cited 28 Aug 2020]. Available: https://www.health.go.ke/cs-health-signs-uhc-scale-up-agreement-with-governors/

21. Mugo I. Nyeri’s chronic disease burden | Nation. 2018 [cited 23 Oct 2020]. Available: https://nation.africa/kenya/healthy-nation/nyeri-s-chronic-disease-burden-117472

22. Kenya National Bureau of Statistics (KNBS). 2019 Kenya Population and Housing Census - Population by County and Sub-County - Kenya Data Portal. 2019 [cited 17 Jul 2020]. Available: https://kenya.opendataforafrica.org/msdpnbc/2019-kenya-population-and-housing-census-population-by-county-and-sub-county?county=1001310-makueni

23. United Nations Office for the Coordination of Humanitarian Affairs S and EA (OCHA R. Kenya Human Development Index per county - Humanitarian Data Exchange. 2015 [cited 21 Oct 2020]. Available: https://data.humdata.org/dataset/kenya-human-development-index-per-county

24. Nyeri County Government. Nyeri County Annual Development Plan. Nyeri, Kenya; 2020.

25. Republic of Kenya. Kenya Health Sector Strategic Plan July 2018 - June 2023. Nairobi, Kenya; 2018.

26. Commission for the Implementation of the Constitution. County Government of Nyeri Department of Health Services Health Facilities Assessment Report. Nyeri, Kenya; 2015.

27. Gathara P. Behind the Makueni Healthcare Revolution. Nairobi: The Elephant; 2018. Available: https://www.theelephant.info/radio/2018/01/11/behind-the-makueni-healthcare-revolution/

28. ThinkWell Gobal. Kenya Knowledge and Learning Brief 1: A Review of Makueni Care. 2019. Available: https://thinkwell.global/wp-content/uploads/2020/02/Makueni-Care-Brief-2019_10_09-Final.pdf

29. Government of Makueni County. County Statistical Abstract Makueni County. Makueni, Kenya; 2020. Available: https://www.knbs.or.ke/?wpdmpro=makueni-county-statistical-abstract
