# Supplementary material for: Household access to non-communicable disease medicines during universal health care roll-out in Kenya: A time series analysis
Source: PLoS One. 2022 Apr 20;17(4):e0266715. doi: 10.1371/journal.pone.0266715 (PMC9020677; doi:10.1371/journal.pone.0266715)
Supplement: S2 Appendix — (DOCX) [file pone.0266715.s008.docx]

**S2 Appendix: Effect of UHC on outcomes of interest for the surveillance sub-sample selected at baseline**

|  | Unadjusted Effect^a^ | | Adjusted Effect^b^ | |
| --- | --- | --- | --- | --- |
|  | **β (95% CI)** | **p-value** | **β (95% CI)** | **p-value** |
| Medicines Available  (N=3,473) | 0.0002 (-0.064, 0.064) | 0.996 | -0.007 (-0.075, 0.060) | 0.831 |
|  | **OR (95% CI)** | **p-value** | **OR (95% CI)** | **p-value** |
| Proportion of Medicines Obtained in Public Hospitals  (N=4,638) | 0.48 (0.31, 0.76) | 0.002 | 0.44 (0.28, 0.69) | 0.00 |
| Proportion of Free Medicines  (N= 4,304) | 1.75 (1.08, 2.82) | 0.022 | 1.47 (0.92, 2.35) | 0.109 |

**^a^ Unadjusted effects: adjusted for time as fixed effects and respondent and County as random effects**

**^b^ Adjusted effects: adjusted for time, County, baseline demographics and baseline NCD diagnosis as fixed effects, and respondent and County as random effects**

Undertaking a post-hoc power analysis, which accounted for correlation within county and within participants over time, we determined that this analysis is powered to detect a 13-percentage point increase in free purchases, given the observed 27% probability of free purchases in the non-UHC group. We ran an adjusted linear probability model and observed an increase of 5 percentage points in free purchases in the UHC group, corresponding to the adjusted OR of free purchases 1.47. Therefore, this analysis is underpowered.
