# Supplementary material for: Household access to non-communicable disease medicines during universal health care roll-out in Kenya: A time series analysis
Source: PLoS One. 2022 Apr 20;17(4):e0266715. doi: 10.1371/journal.pone.0266715 (PMC9020677; doi:10.1371/journal.pone.0266715)
Supplement: S3 Appendix — (DOCX) [file pone.0266715.s009.docx]

**S2 Appendix: Proportion of NCD medicines obtained in public health centers/clinics**

**Table A: Effect of UHC on medicines obtained in public health centers/clinics**

|  | Unadjusted Effect^a^  (N=6,436) | | Adjusted Effect^b^  (N=6,424) | |
| --- | --- | --- | --- | --- |
|  | **OR (95% CI)** | **p-value** | **OR (95% CI)** | **p-value** |
| Proportion of Medicines Obtained in Public Health Centers/Clinics | 2.56 (1.63, 4.02) | 0.00 | 2.49 (1.56, 3.97) | 0.00 |

**^a^ Unadjusted effects: adjusted for time as fixed effects and respondent and County as random effects**

**^b^ Adjusted effects: adjusted for time, County, baseline demographics and baseline NCD diagnosis as fixed effects, and respondent and County as random effects**

**Fig A: Proportion of free medicines in public hospitals in “Switched to UHC” group**

**Fig B: Proportion of free medicines in public clinics/health centers in “Switched to UHC” group**
